# Optimization of tetramycin production in *Streptomyces ahygroscopicus* S91

**DOI:** 10.1186/s13036-021-00267-4

**Published:** 2021-05-22

**Authors:** Guang Chen, Mengqiu Wang, Xianpu Ni, Huanzhang Xia

**Affiliations:** School of Life Science and Biopharmaceutics, Shenyang Pharmaceutical University, No.103 Wenhua Road, Shenyang, Liaoning China

**Keywords:** Tetramycin, *Streptomyces ahygroscopicus*, Polyene macrolide antibiotics, Cytochrome P450 monooxygenase, Metabolic engineering

## Abstract

**Background:**

Tetramycin is a 26-member tetraene antibiotic used in agriculture. It has two components, tetramycin A and tetramycin B. Tetramycin B is obtained by the hydroxylation of tetramycin A on C4. This reaction is catalyzed by the cytochrome P450 monooxygenase TtmD. The two components of tetramycin have different antifungal activities against different pathogenic fungi. Therefore, the respective construction of high-yield strains of tetramycin A and tetramycin B is conducive to more targeted action on pathomycete and has a certain practical value.

**Results:**

*Streptomyces ahygroscopicus* S91 was used as the original strain to construct tetramycin A high-yield strains by blocking the precursor competitive biosynthetic gene cluster, disrupting tetramycin B biosynthesis, and overexpressing the tetramycin pathway regulator. Eventually, the yield of tetramycin A in the final strain was up to 1090.49 ± 136.65 mg·L^− 1^. Subsequently, TtmD, which catalyzes the conversion from tetramycin A to tetramycin B, was overexpressed. Strains with 2, 3, and 4 copies of *ttm*D were constructed. The three strains had different drops in tetramycin A yield, with increases in tetramycin B. The strain with three copies of *ttm*D showed the most significant change in the ratio of the two components.

**Conclusions:**

A tetramycin A single-component producing strain was obtained, and the production of tetramycin A increased 236.84% ± 38.96% compared with the original strain. In addition, the content of tetramycin B in a high-yield strain with three copies of *ttm*D increased from 26.64% ± 1.97 to 51.63% ± 2.06%.

**Supplementary Information:**

The online version contains supplementary material available at 10.1186/s13036-021-00267-4.

## Background

Secondary metabolites produced by microorganisms have various physiological activities and are important sources of natural drugs [1]. In recent years, with the development of biotechnologies, metabolic engineering based on recombinant DNA technology has been increasingly applied to increase the yield of secondary metabolites, bringing advantages in improving the content of metabolic components and producing non-natural new compounds [2]. The primary methods of metabolic engineering are as follows. (1) Changing the distribution of precursor metabolic fluxes: the precursors used to synthesize secondary metabolites are primarily derived from the primary metabolism of microorganisms and are one of the key factors that determine the yield of secondary metabolites. By inactivating the precursor competing biosynthetic gene clusters and changing the metabolic fluxes of the precursor, the yield of the target product can be increased effectively [3–5]. (2) Blocking or overexpressing of the structural genes: Through the inactivation or overexpression of some specific structural genes in the biosynthesis gene cluster (BGC) of secondary metabolites, the metabolic pathway can be terminated at an intermediate product or the metabolite can be further metabolized into a final product to improve the proportions of the secondary metabolites [6, 7]. (3) Expressing and blocking regulatory genes: The biosynthesis of secondary metabolites is typically regulated by specific regulators within its BGC, and sometimes by a pleiotropic regulator. Pathway-specific regulatory factors can activate or inhibit the transcription of structural genes. Overexpression of positive regulators or inhibition of negative regulators can be beneficial for the accumulation of secondary metabolites [8–11]. In addition, (i) increasing the resistance, (ii) the use of genome rearrangement technology, and (iii) using heterologous hosts or the expression of BGCs in production strains [12] are also common methods of metabolic engineering.

Polyene macrolides are polyketones that include pimaricin, nystatin, amphotericin, and tetramycin. Polyene macrolides are primarily used in the treatment of clinical fungal infections, food preservation, and agricultural fungal disease prevention. In addition, polyene macrolides show antitumor, antiviral, antiprotozoal and immunosuppressive activities with great antibacterial potential [13–16]. Tetramycin is a 26-member tetraene macrolide that can be produced by *Streptomyces noursei, Streptomyces hygrospino*s*u*s, and *Streptomyces ahygroscopicus* [17]. As a biological fungicide, tetramycin is primarily used for the prevention and control of leaf spot disease, rice blast disease, and gray mold [18–20]. Tetramycin consists of two components, tetramycin A (TA) and tetramycin B (TB). However, the antifungal activities of the two components are different. TA prefers to prevent *Saccharomyces cerevisiae* and *Aspergillus flavus*, whereas TB has stronger effects on *Fusarium solani*, *Penicillium notatum*, and *Scopulariopsis* [21, 22]. Therefore, it is of potential value to obtain high-yield strains of each of the two components for industrial production.

The biosynthetic pathway and regulatory factors of tetramycin have been studied [23–25]. Tetramycin biosynthesis follows the common polyene antibiotics biosynthesis pathway, with small molecular carboxylic acids (acetyl-CoA, malonyl-CoA, methylmalonyl-CoA, and ethylmalonyl-CoA) as TA precursors. Tetramycinolide is formed by the polyketide synthase pathway (PKS), TA is formed through carboxylation and glycosylation by post-PKS tailoring, and TB is formed from TA through C4 hydroxylation (Fig. S1). In this study, the production of tetramycin is improved by means of metabolic engineering using *Streptomyces ahygroscopicus* S91(GCMCC 4.7082) as the original strain. The genome of *S. ahygroscopicus* S91 contains various BGCs and produces several of secondary metabolites, including tetramycin, nystatin, anisomycin, and toyocamycin. Tetramycin and nystatin are polyene macrolide antibiotics that share the common precursors acetyl-CoA, malonyl-CoA, and methylmalonyl-CoA in the biosynthesis process. By blocking the biosynthesis of nystatin, the metabolic fluxes of the precursors are redirected, improving the production of tetramycin. TB is the hydroxylation product of TA, which is converted by the cytochrome P450 monooxygenase TtmD. Blocking *ttm*D expression can eliminate the conversion to TB. Hence, TA is obtained separately. The overexpression of *ttm*D is beneficial to increase the conversion efficiency of TA, and the proportion of TB can be raised. Cui et al. conducted a study on the regulatory mechanism of tetramycin biosynthesis and found that there were four pathway-specific regulators (TtmRI, TtmRII, TtmRIII, and TtmRIV) in the tetramycin BGC. TtmRIV belongs to the positive regulatory factors of the PAS-LuxR family, and its overexpression improved the TA yield [25].

By using the metabolic engineering for the biosynthesis of secondary metabolites, the tetramycin-producing strain, *S. ahygroscopicus* S91, is expected to optimize the composition of tetramycin and enrich the metabolic engineering application of polyene macrolide antibiotics.

## Results

### Construction of the tetramycin-producing strain by disrupting the biosynthesis of nystatin

**Fig. 1:**
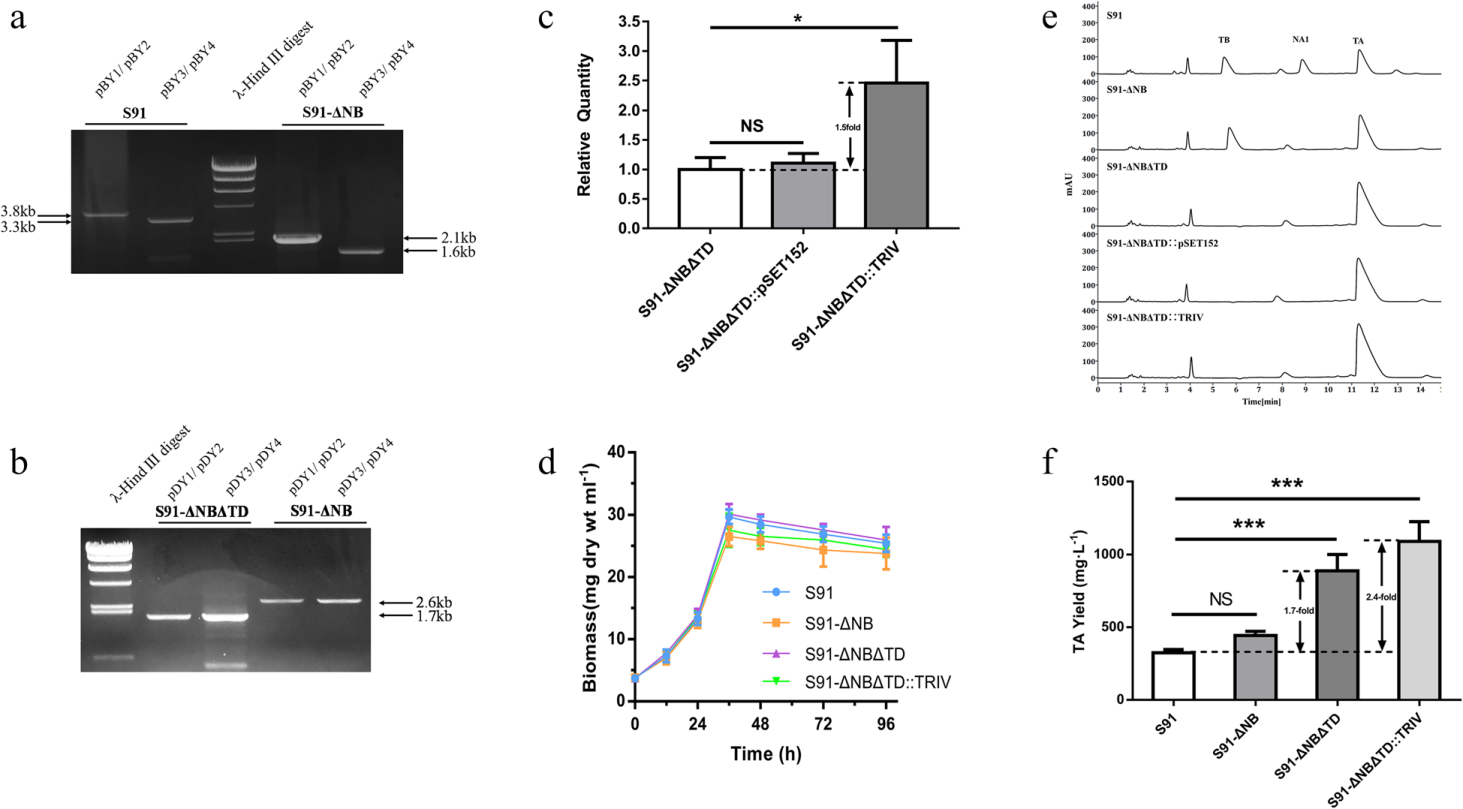
Improved production of TA. **a** Verification of the *nys*B disruption in the S91-ΔNB strain. The PCR products of pBY1/pBY2 and pBY3/pBY4 with S91 total DNA were 3.8 kb and 3.3 kb, respectively, and with S91- ΔNB total DNA were 2.1 kb and 1.6 kb, respectively. A 1714 bp DNA fragment in the *nys*B framework was deleted in the S91-ΔNB strain. **b** Verification of the *ttm*D disruption in the S91-ΔNBΔTD strain. The PCR products of pDY1/pDY2 and pDY3/pDY4 with S91-ΔNB total DNA were both 2.6 kb, and with S91-ΔNBΔTD total DNA were both 1.7 kb. An 881 bp DNA fragment in the *ttm*D framework was deleted in the S91-ΔNBΔTD strain. **c** Transcriptional analysis of the *ttm*RIV in the S91-ΔNBΔTD, S91-ΔNBΔTD::pSET152 and S91-ΔNBΔTD::RIV strains using qRT-PCR. The *ttm*RIV was under the control of the *hrdB* promoter. The relative values for the *ttm*RIV in the S91-ΔNBΔTD strain was assigned as 1, with *hrd*B as the internal control. **d** The biomass of *S. ahygroscopicus* S91 and its mutants. **e** The HPLC analysis of the fermentation products in *S. ahygroscopicus* S91 and its mutants. The LC-MS analysis of tetramycin A (TA), tetramycin B (TB), and nystatin A1 (NA1) are shown in Fig. S2. **f** Production of tetramycin A in *S. ahygroscopicus* S91 and its mutants. Error bars depict standard deviation of three replicates. ***P<0.001, **P<0.01, *P<0.05, and “ns”means not significant

The primary intracellular fermentation products of *S. ahygroscopicus* S91 are tetramycin (TA and TB) and nystatin (NA1) (Fig. S2). Acetyl-CoA, malonyl-CoA, and methylmalonyl-CoA were the precursors used in the biosynthesis process of these products. Therefore, an increase in the yield of tetramycin was expected by disrupting the biosynthesis of nystatin and redirecting the fluxes of precursors to the biosynthetic pathway of tetramycin. *NysB*, the first extension module of PKS in nystatin BGC, was chosen to be disrupted to minimize the loss of precursors. A 1714 bp DNA fragment in the *nys*B framework was deleted to construct the nystatin disruption strain S91-ΔNB (Fig. 1a, Fig. S3). The fermentation results of S91-ΔNB showed that S91-ΔNB no longer produced NA1. In addition, the TA and TB contents increased from 35.61% ± 1.80 and 21.31% ± 1.18 to 51.59% ± 3.90 and 26.64% ± 1.97%, respectively. The total production of TA and TB increased from 517.49 ± 24.72 mg·L^− 1^ to 657.25 ± 29.77 mg·L^− 1^, respectively (Fig. 1e, f, Tab. S1).

### Construction of the TA single-component high-yield strain

TA and TB have different antifungal effects on different fungi, and TA has stronger antifungal effects on *Saccharomyces cerevisiae* and *Aspergillus flavus* than TB. TB is hydroxylated TA, and the reaction is catalyzed using the cytochrome P450 monooxygenase TtmD. A TA single-component strain was expected to be obtaind by blocking the *ttm*D. *Ttm*D is located upstream of the PKS *ttm*S1, and the two genes have opposite polarities. To avoid influences on the *ttm*S1 transcription, an 881 bp fragment within the framework of *ttm*D was deleted to generate S91-ΔNBΔTD (Fig. 1b, Fig. S4). The recombinant strain S91-ΔNBΔTD produced only TA. The total yield of tetramycin in the S91-ΔNBΔTD strain was 33.18% ± 12.16% higher than that of S91-ΔNB. The yield of TA increased significantly, from 443.22 ± 29.19 mg·L^− 1^ to 888.62 ± 111.98 mg·L^− 1^ (an increase of 100.49% ± 15.57%) (Fig. 1e, f, Tab. S1).

Previous work demonstrated that *ttm*RIV was a positive regulatory gene in tetramycin BGC, and its overexpression improved the TA yield. Therefore, the addition of the copy of *ttm*RIV in the TA single-component producing strain was expected to further improve the TA yield. Using the *S. ahygroscopicus* S91 total DNA as a template, the 624 bp *ttm*RIV fragment was obtained by PCR and inserted into pSET152 to construct an overexpression plasmid. The *ttm*RIV was under the control of the *hrdB* promoter. Then S91-ΔNBΔTD strain was transformed to obtain S91-ΔNBΔTD::TRIV and S91-ΔNBΔTD::pSET152. The qRT-PCR analysis showed that the expression level of *ttm*RIV in the overexpression strain S91-ΔNBΔTD::TRIV was nearly 1.5 times higher than that in S91-ΔNBΔTD (Fig. 1c). The fermentation results showed that the TA yield in S91-ΔNBΔTD::TRIV reached 1090.49 ± 136.65 mg·L^− 1^, which was 22.75% ± 2.40% higher than that in S91-ΔNBΔTD (Fig. 1e, f, Tab. S1). The biomass of *S. ahygroscopicus* S91 and its mutants was shown in Fig. 1d.

### Construction of the TB high-yield strains by using the overexpression of *ttm*D

**Fig. 2:**
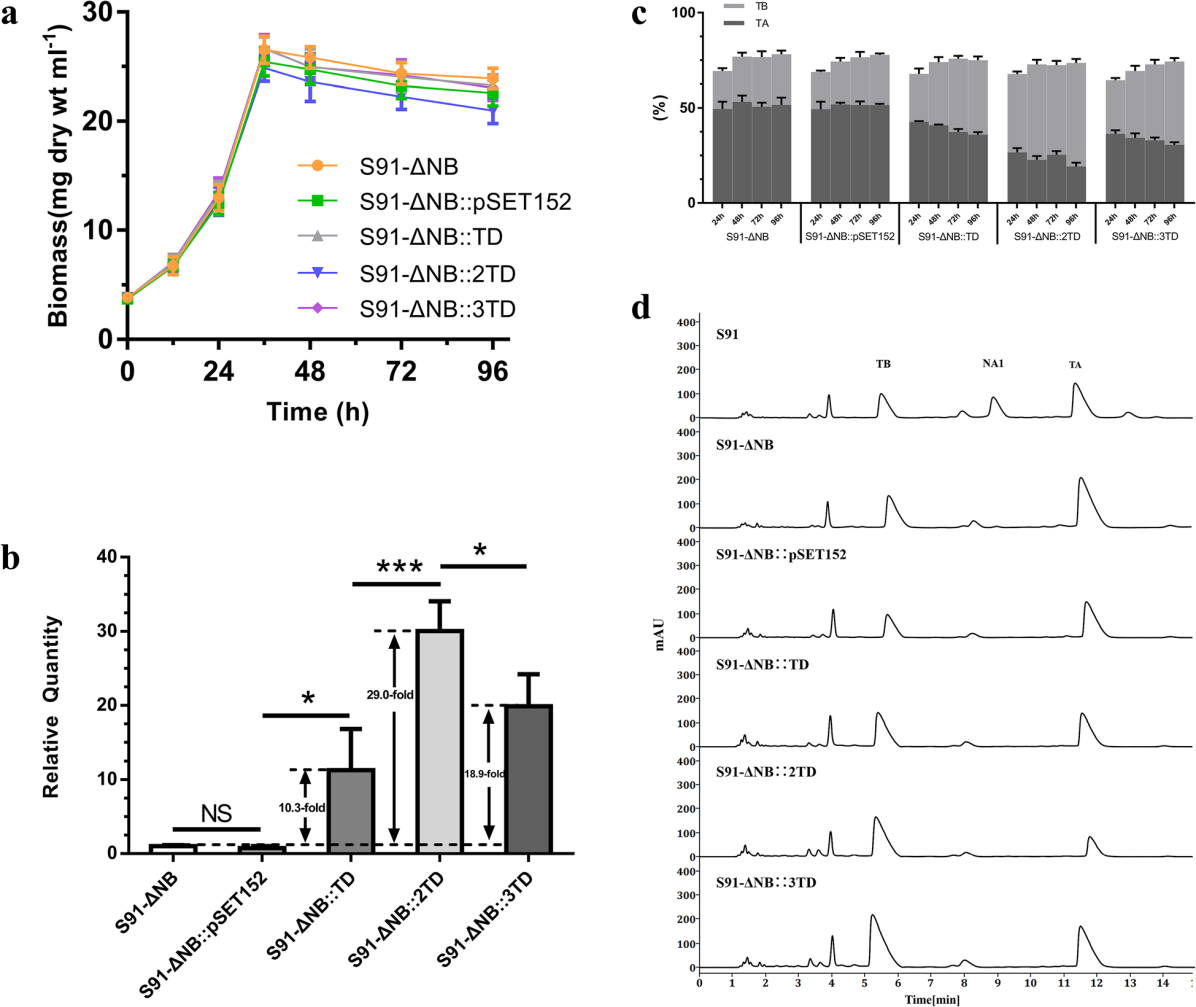
Improved production of TB. **a** The biomass of *S. ahygroscopicus* S91-ΔNB and the multicopy *ttm*D strains. The S91-ΔNB::TD, S91-ΔNB::2TD, and S91-ΔNB::3TD strains have two copies, three copies, and four copies of *ttm*D, respectively. **b** Transcriptional analysis of the *ttm*D in S91-ΔNB and the multicopy *ttm*D strains using qRT-PCR. The *ttm*D was under the control of the *hrdB* promoter. The relative values for the *ttm*D in the S91-ΔNB strain was assigned as 1, with *hrd*B as the internal control. **c** The content analysis of TA and TB in S91-ΔNB and the multicopy *ttm*D strains at 24 h, 48 h, 72 h, and 96 h. **d** The HPLC analysis of fermentation products in S91-ΔNB and the multicopy *ttm*D strains. Error bars depict standard deviation of three replicates. ***P<0.001, **P<0.01, *P<0.05, and “ns”means not significant

TB is produced by the hydroxylation of TA on C4, which is catalyzed by the cytochrome P450 monooxygenase TtmD. Therefore, it was hypothesized that the production of TB could be improved by increasing the copies of *ttm*D in the S91-ΔNB strain. Single-copy, two-copy, and three-copy *ttm*D plasmids were constructed based on the pSET152 vector, and three multicopy plasmids and pSET152 were introduced into the S91-ΔNB strain, S91-ΔNB::TD, S91-ΔNB::2TD, and S91-ΔNB::3TD, and S91-ΔNB::pSET152 strains were obtained. The *ttm*D was also under the control of the *hrdB* promoter. The expression of *ttm*D in the multicopy *ttm*D strains was analyzed using qRT-PCR. Compared with the original strain, the expression levels of *ttm*D in the recombinant strains increased 10.3-, 29.0-, and 18.9-fold (Fig. 2b).

Subsequently, the tetramycin content in the recombinant strains during the fermentation period were observed (Fig. 2c). The total tetramycin content was slightly lower within 24 h, and each strain ranged from 64 to 69%. As the fermentation time increased, the tetramycin yield increased and the content increased slightly. After 48 h, the content of the two components in each strain was within the range of 73–77%. The TA content of S91-ΔNB was basically stable at each fermentation time, while the TB content increased slightly (6.69% ± 0.43%). The proportion of TA from 24 h to 96 h decreased by 6.81% ± 1.01, 7.36% ± 0.27, and 5.78% ± 0.56% in the multicopy *ttm*D recombinant strains. TB showed an increasing trend, and the proportion of TB at 96 h was 14.04% ± 0.92, 13.20% ± 0.73, and 15.56% ± 0.77% higher. The proportion of TB increased with the proportion of TA dropped, which indicated that TA gradually transformed into TB during the fermentation process. The TB content in S91-ΔNB::2TD increased to 51.63% ± 2.06%, and the yield reached 370.79 ± 14.79 mg·L^− 1^, nearly twice as high as in the original strain. The TB content in S91-ΔNB::3TD increased to 44.76% ± 1.90%, and the yield reached 533.59 ± 22.65 mg·L^− 1^ (Fig. 2d, Tab. S1). The biomass of S91-ΔNB and its mutants was shown in Fig. 2a.

## Discussion

Tetramycin is an important fungicide, and its strains are known to be associated with the production of other polyene macrolide antibiotics, such as nystatins and tetrins [22]. It is difficult to separate tetramycin from the by-products of its producing strains owing to their similar synthesis pathways and common precursors. Metabolic engineering is an effective and practical approach to reducing the quantity of by-products and improving the yield of tetramycin. In this study, the competing pathway of nystatin biosynthesis was blocked in the original strain *S. ahygroscopicus* S91, mainly generating the tetramycin strain S91-ΔNB. Relative to the original strain, S91-ΔNB showed no significant improvement in the total yield of tetramycin, as the reduction in nystatin was almost equivalent to the increase in tetramycin. However, in the subsequent experiment, when the cytochrome P450 monooxygenase TtmD was inactive in the recombinant strain S91-ΔNBΔTD, the total yield of tetramycin increased 33.18% ± 12.16% over S91-ΔNB. TtmD is responsible for the conversion from TA to TB, which is similar to its counterpart NysL, AmphL, and PimD, performing the final biosynthetic step on polyene. These four enzymes catalyze different chemical reactions: hydroxylation of the C5 in tetramycin (TtmD), hydroxylation of the C10 in nystatine (NysL) [26], hydroxylation of the C8 in amphotericin (AmphL) [27], and epoxidation of the C4-C5 double bond in pimaricin (PimD) [28]. All of these reactions require NADPH as a reducing factor. In the biosynthesis of polyenes and other polyketides, NADPH is usually consumed in the reduction of enoylreductase (ER) of PKS and the tailoring modification of macrolides [29, 30]. Disruption of *ttm*D in S91-ΔNBΔTD decreased NADPH consumption, and more NADPH was redirected into biosythesis of PKS to improve the yield of TA to some extent. For the same reason, an excessive overexpression of *ttm*D may weaken the biosynthesis of PKS. Even though the proportion of TA and TB showed the greatest optimization in the three-copy *ttm*D strain S91-ΔNB::2TD, the total yield of tetramycin was not the highest.

Regarding the overexpression of *ttm*RIV and *ttm*D, the *hrdB* promoter was used to control the transcription. Generally, the introduction of a strong promoter is an effective strategy for improving product yield and activating cryptic gene clusters [31]. In our previous study on *ttm*D, three promoters, including the *ttm*D native promoter, the ermE* promoter, and the *hrdB* promoter, were separately introduced into the *ttm*D disruption strain S91-ΔTD and the efficiency of expression was assessed. We found the *hrdB* promoter to be the most efficient, and this was confirmed in the multicopy *ttm*D strains. Regarding *ttm*RIV, the *hrdB* promoter fostered efficiency to a much lower extent than *ttm*D, so the improvement in the yield of TA was limited. Currently, stronger promoters, such as *kasO*p* are used to overexpress the rate-limiting biosynthetic genes in some streptomyces, and the yield of products improved significantly [32, 33]. In this way, this method offers the opportunity to further improve the TA yield by overexpression of *ttm*RIV under these promoters and by introducing multiple copies of *ttm*RIV.

Many other metabolic engineering approaches can also improve the yield of both TA and TB. In these strategies, increasing the supply of precursors can be direct and effective. Generally, the supply of various acyl-CoAs is the limiting factor in the biosynthesis of polyketides. It can be overcome by overexpressing the genes encoding the key enzymes such as acetyl-CoA carboxylase (ACC), propionyl-CoA carboxylase (PCC), and crotonyl-CoA carboxylase/reductase (CCR) [34–36]. ACC catalyzes the conversion from acetyl-CoA to malnonyl-CoA, PCC plays a key role in increasing methylmalonyl-CoA, and CCR catalyzes the conversion from crotonyl-CoA to ethylmalonyl-CoA. Ten malonyl-CoAs, one methylmalonyl-CoA, and one ethylmalonyl-CoA are involved in the biosynthesis of tetramycin, so the overexpression of these genes is expected to increase the intracellular pool of precursors and improve the yield of tetramycin. Recently, branched chain amino acid (BCAA) metabolism has been shown to be the bottleneck of the biosynthesis of some polyketides [37, 38]. BCAA degradation pathway can supply various acyl-CoAs. Manipulation on the key enzymes of BCAA metabolism, such as branched chain α-keto acid dehudrogenase (BCDH) and acyl-CoA dehydrogenase (ACAD), can also improve the yield of tetramycin.

As mentioned above, cytochrome P450 monooxygenases catalyze hydroxylation (TtmD, NysL, and AmphL) or epoxidation (PimD) of polyene macrolides. TtmD shows 59% sequence identity with NysL, 56% with AmphL and 68% with PimD. It has been reported [39] that these enzymes are not as substrate-specific as previously thought. *Ttm*D, *nys*L, and *amph*L were separately introduced into a *Streptomyces natalensis* Δ*pim*D strain whose fermentation product was 4,5-desepoxypimamycin (DEP). The results showed that TtmD can convert DEP into pimaricin, indicating that TtmD can perform hydroxylation and epoxidation. NysL catalyzes the conversion of DEP to C6-OH-DEP, a new derivative. However, AmphL does not catalyze the conversion. A similar experiment was performed in S91-ΔNBΔTD. Phylogenetic tree analysis and homology modeling results show that TtmD and PimD have considerable structural similarity. For this reason, *pim*D was introduced into S91-ΔNBΔTD as *ttm*D. Although PimD was expressed in S91-ΔNBΔTD, TA was not converted to TB. Furthermore, no other new products were detected (not shown). These results indicate that the substrate specificity of PimD is more strict than that of TtmD. PimD recognizes double bond at C4-C5 of DEP, whereas the counterpart of TA is a saturated bond. The saturated bond is caused by the inactivation DH11 in PKS of tetramycin BGC. It is reasonable to assume that domain swapping, by some active agents such as DH11 in PKS of pimaricin BGC, could generate a double bond at C4-C5 which is subsequently epoxidized by TtmD to form new tetramycin derivatives.

## Conclusions

High-yield strains that produced TA and TB were obtained in this study. During the process of constructing the high yield strain of TA, nystatin BGC was first blocked, and yield of tetramycin was increased to 667.25 ± 29.77 mg·L^− 1^; hence, the TA yield was 443.22 ± 29.19 mg·L^− 1^. Then, *ttm*D was blocked, the TA yield was increased to 888.62 ± 111.98 mg·L^− 1^. After that, the positive regulatory gene of tetramycin BGC was overexpressed. Hence, the TA yield was further increased to 1090.49 ± 136.65 mg·L^− 1^.

In the construction of TB high-proportion strain, strains with different copy numbers of *ttm*D were constructed to increase the content of TB. The results showed that the TB content in the strain with three copies of *ttm*D was the highest, increasing from 26.64% ± 1.97 to 51.63% ± 2.06%.

## Methods

### Strains, plasmids, medium, and cultivation conditions

The strain *S. ahygroscopicus* S91 was used as the initial strain, which had been deposited at the China General Microbiology Culture Collection Center (accession No. CGMCC 4.7082), Institute of Microbiology, the Chinese Academy of Science. The other plasmids and primers used in this study are listed in Table S2.

*S. ahygroscopicus* S91 and its mutants were maintained on Gause’s synthetic agar medium (2% soluble starch, 0.1% Beef extract, 0.1% KNO_3_, 0.05% MgSO_4_·7H_2_O, 0.05% K_2_HPO_4_·3H_2_O, 0.05% NaCl, 0.001% FeSO_4_·7H_2_O, 2.5% agar, and pH 7.2) at 28 °C. *E. coli* strains were cultured in the LB broth or agar at 37 °C. 2 × YT medium was used for the germination of *Streptomyces* spores, and MS was used for co-culture of *Streptomyces* and *E·coli* in conjugation. For fermentation, the seed medium contained 2% glucose, 0.6% peptone, 0.6% yeast extract, 1% NaCl, at a pH of 7.2. The fermentation medium contained 2% corn powder, 0.8% corn starch, 3% soya bean, 3% glucose, 0.02% NaCl, 0.02% MgSO_4_·7H_2_O, 0.02% K_2_HPO_4_·3H_2_O, 0.02% FeSO_4_·7H_2_O, 0.25% (NH_4_)_2_SO_4_, 0.5% CaCO_3_, at a pH of 7.2. Then 2% agar was added to this for the solid fermentation medium used to culture the mycelia during the process of RNA extraction.

### Inactivation of *nys*B

To disrupt the biosynthesis of nystatin, the genomic DNA of *S. ahygroscopicus* S91 was used as a template, and the primers NB-UF/NB-UR and NB-DF/NB-DR were used for the PCR. The 1452 bp upstream homologous fragment, *NBU*, and the 1456 bp downstream homologous fragment, *NBD*, were obtained using PCR amplification. After sequencing verification, they were jointly ligated to the pKC1139 vector between the *Hin*dIII and *Bam*HI restriction sites, and the blocking plasmid pDNB was constructed (Fig. S3a). After that, pDNB was transferred into *E. coli* ET12567 (pUZ8002) and introduced into *S. ahygroscopicus* S91 by conjugation, and apramycin-resistant strains were selected for subculture. The stable apramycin-sensitive strains were screened after three generations of relaxed culture. The nystatin disruption strain, S91-ΔNB, was obtained. Two validation primer pairs (pBY1/pBY2 and pBY3/pBY4) were used for the double crossover validation using PCR amplification (Fig. S3b, c).

### Inactivation of *ttm*D

The primers TD-UF/TD-UR and TD-DF/TD-DR were used to amplify the 1538 bp upstream homologous fragment, *TDU*, and the 1005 bp downstream homologous fragment, *TDD*, of *ttm*D. After sequencing verification, they were jointly ligated to the pKC1139 vector between the *Hin*dIII and *Eco*RI restriction sites, and the blocking plasmid, pDTD, was constructed (Fig. S4a). After that, pDTD was transferred into *E. coli* ET12567 (pUZ8002) and introduced into *S. ahygroscopicus* S91-ΔNB by conjugation. The apramycin-resistant strains were selected for subculture, and the stable apramycin-sensitive strains were screened after three generations of relaxed culture. The *ttmD* deletion strain, S91-ΔNBΔTD, was then obtained. Two validation primer pairs (pDY1/pDY2 and pDY3/pDY4) were used for the double crossover validation using PCR amplification (Fig. S4b, c).

### Cloning and overexpression of *ttm*RIV

The primers, TRIV-F and TRIV-R, were used to amplify the 624 bp *ttm*RIV gene fragment. The *ttm*RIV fragment was digested using *Nco*I and *Xho*I and ligated to pPT2925, which was digested using the same enzymes, to generate the recombinant plasmid pTRIV. pTRIV was digested using *Bgl*II and a 1.5 kb fragment containing the *hrdB* promoter, *ttm*RIV, and the *T*_*0*_ terminator was ligated to pSET152. pSET152 was digested using *Bam*HI and dephosphorylated to construct overexpression plasmid pETRIV (Fig. S5a). After this, pETRIV was transferred into *E. coli* ET12567 (pUZ8002) and introduced into *S. ahygroscopicus* S91-ΔNBΔTD by conjugation, and the apramycin-resistant strains were selected. The strain S91-ΔNBΔTD::TRIV with two copies *ttm*RIV was obtained. The primers, PB-1/TRIV-R, were used for verification (Fig. S5b).

### Cloning and overexpression of *ttm*D

The 1191 bp *ttm*D fragment, *TD*, was amplified from *S. ahygroscopicus* S91 genomic DNA using the primers TD-F and TD-R and ligated to the plasmid pPT2925, digested using *Nco*I and *Xho*I, to generate the recombinant plasmid pPTD. The 2.1 kb fragment containing the *hrdB* promoter, *TD*, and the *T*_*0*_ terminator (*P*_*hrdB*_-*TD*-*T*_*0*_) was obtained using *Bgl*II digestion and ligated to pSET152, which was digested with *Bam*HI and dephosphorylated to construct the overexpression plasmid pETD.

The 2.1 kb *P*_*hrdB*_-*TD*-*T*_*0*_ fragment was obtained when pPTD was digested using *Eco*RI and *Spe*I, and then ligated to the pPTD between the *Eco*RI and *Xba*I restriction sites to generate the recombinant plasmid p2PTD. Then the p2PTD was digested using *Bgl*II, and the 4.2 kb fragment containing two copies of *P*_*hrdB*_-*TD*-*T*_*0*_ was ligated to pSET152, which was digested using *Bam*HI and dephosphorylated to construct the overexpression plasmid p2ETD, which contained two copies of *ttm*D (Fig. S6a).

The construction of p3PTD with three copies of *ttm*D was similar to the construction of p2PTD in which the 2.1 kb *P*_*hrdB*_-*TD*-*T*_*0*_ fragment was ligated to p2PTD. In addition, the overexpression plasmid p3ETD was obtained from p3PTD in the same manner as p2ETD as described above.

All of the three plasmids pETD, p2ETD, and p3ETD were transferred into *E. coli* ET12567 (pUZ8002) and introduced into *S. ahygroscopicus* S91-ΔNB by conjugation, and the apramycin-resistant strains were selected. Three multicopy *ttm*D strains were obtained. The primers, PB-1/TD-R, were used for verification (Fig. S6b).

### Purification of tetramycin and detection conditions using HPLC

*S. ahygroscopicus* S91 and its mutants were cultured in seed medium at 28 °C for 24 h. The seed cultures were inoculated into fermentation medium to 10% (v/v) and cultured at 28 °C for 96 h. Then the mycelia was harvested and extracted with methanol.

The extracts were subjected to HPLC analysis (Agilent series 1260, Agilent Technologies, USA) under the following conditions: column: Agilent EC-C18 column (150 × 4.6 mm, 4 μm); column temperature: 34 °C; wave length: 304 nm; flow rate: 1 mL·min^− 1^; injection volume: 5 μL; mobile phase: water (solvent A) and methanol: formic acid = 60: 0.1 (solvent B). Elution was performed as follows: 40% A: 60% B,0–5 m; down to 35% A: 65% B, 5–8 m; 35% A: 65%, B 8–18 m.

### RNA isolation and the qRT-PCR analysis

*S. ahygroscopicus* S91 and its mutants were cultured on a solid fermentation medium for 48 h. Then the mycelia was harvested, and the total RNA were isolated using the Ultrapure RNA Kit (DNase I) (Cwbio). cDNA was reverse transcripted using the PrimeScript™ RT Reagent Kit (TaKaRa). The qRT-PCR analysis was performed using the MightyAmp™ for Real Time (SYBR®Plus) (TaKaRa). The relative mRNA levels were analyzed using the 2^−ΔΔCt^ method, with the housekeeping gene *hrd*B as an internal reference. The *hrd*B was amplified using the primers PB-RT-1 and PB-RT-2. The *ttm*RIV was amplified using the primers RIV-RT-1 and RIV-RT-2. The *ttm*D was amplified using the primers TD-RT-1 and TD-RT-2.

## Supplementary Information

**Additional file 1: Figure S1.** Biosynthesis of tetramycin.

**Additional file 2: Figure S2.** LC-MS analysis of tetramycin and nystatin in *Streptomyces ahygroscopicus* S91. a. The HPLC profile of *Streptomyces ahygroscopicus* S91 fermentation products; b. The UV absorption spectra and MS of tetramycin B; c. The UV absorption spectra and MS of nystatin; d. The UV absorption spectra and MS of tetramycin A.

**Additional file 3: Figure S3.** Inactivation of *nys*B in *S.ahygroscopicus* S91. a. Construction of the recombinant plamid pDNB; b. Double crossover validation of the recombinant strain S91-ΔNB; c.Verification of sequencing in the recombinant strain S91-ΔNB.

**Additional file 4: Figure S4.** Inactivation of *ttm*D in *S.ahygroscopicus* S91-ΔNB. a. Construction of the recombinant plasmid pDTD; b. Double crossover validation of the recombinant strain S91-ΔNBΔTD; c. Verification of sequencing in the recombinant strain S91-ΔNBΔTD.

**Additional file 5: Figure S5.** Cloning and overexpression of *ttm*RIV. a. Construction of the recombinant plamid pETRIV; b. PCR analysis of the recombinant strain S91-ΔNBΔTD::TRIV, M. DL2000 (2.0k, 1.0k, 0.75k, 0.5k, 0.25k, 0.1k), 1. S91-ΔNBΔTD::pETRIV/PB-1& TRIV-R (1.1k), 2. S91-ΔNBΔTD/PB-1&TRIV-R, 3. S91-ΔNBΔTD::pSET152/PB-1&TRIV-R.

**Additional file 6: Figure S6.** Cloning and overexpression of *ttm*D. a. Construction of the recombinant plamid p2ETD; b. PCR analysis of the multicope *ttm*D recombinant strains, M. DL2000, 1. S91-ΔNB::pETD/PB-1&TD-R (1.7k), 2. S91-ΔNB::p2ETD/PB-1&TD-R (1.7k), 3. S91-ΔNB::p3ETD/PB-1&TD-R (1.7k), 4. S91-ΔNB/PB-1&TD-R, 5. S91-ΔNB::pSET152/PB-1&TD-R.

**Additional file 7: Table S1.** Production analysis in *S.ahygroscopicus* S91 and its mutants.

**Additional file 8: Table S2.** Strains, plasmids and primers used in this study.

## Data Availability

The DNA sequence of the tetramycin BGC is available to the public in NCBI under accession number: JX827252.1.

## Abbreviations

PKS: Polyketide synthase
TA: Tetramycin A
TB: Tetramycin B
NA1: Nystatin
BGCs: Biosynthetic gene clusters
