# Supplementary figures and images for: Optimization of tetramycin production in Streptomyces ahygroscopicus S91
Source: J Biol Eng. 2021 May 22;15:16. doi: 10.1186/s13036-021-00267-4 (PMC8141235; doi:10.1186/s13036-021-00267-4)

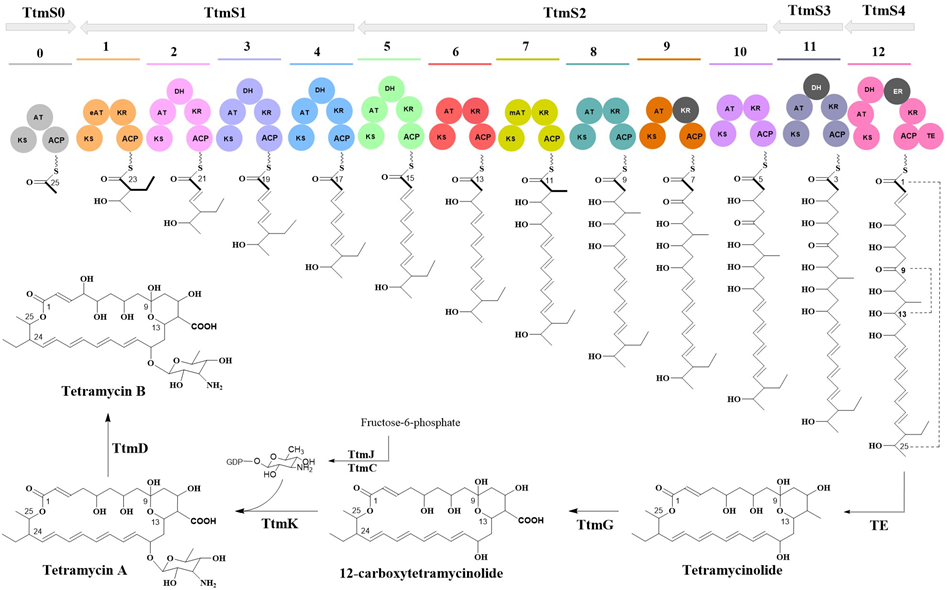


**Figure S1 Biosynthesis of tetramycin**

Supplement: Supplementary file 1 — Additional file 1: Figure S1. Biosynthesis of tetramycin. [file 13036_2021_267_MOESM1_ESM.docx]
