# Supplementary material for: Optimization of tetramycin production in Streptomyces ahygroscopicus S91
Source: J Biol Eng. 2021 May 22;15:16. doi: 10.1186/s13036-021-00267-4 (PMC8141235; doi:10.1186/s13036-021-00267-4)
Supplement: Supplementary file 2 — Additional file 2: Figure S2. LC-MS analysis of tetramycin and nystatin in Streptomyces ahygroscopicus S91. a. The HPLC profile of Streptomyces ahygroscopicus S91 fermentation products; b. The UV absorption spectra and MS of tetramycin B; c. The UV absorption spectra and MS of nystatin; d. The UV absorption spectra and MS of tetramycin A. [file 13036_2021_267_MOESM2_ESM.docx]

**Figure S2 Descriptions**

**Fig. S2** LC-MS analysis of tetramycin and nystatin in *Streptomyces ahygroscopicus* S91

a. The HPLC profile of *Streptomyces ahygroscopicus* S91 fermentation products; b. The UV absorption spectra and MS of tetramycin B; c. The UV absorption spectra and MS of nystatin; d. The UV absorption spectra and MS of tetramycin A.

**Figure S2a**

**
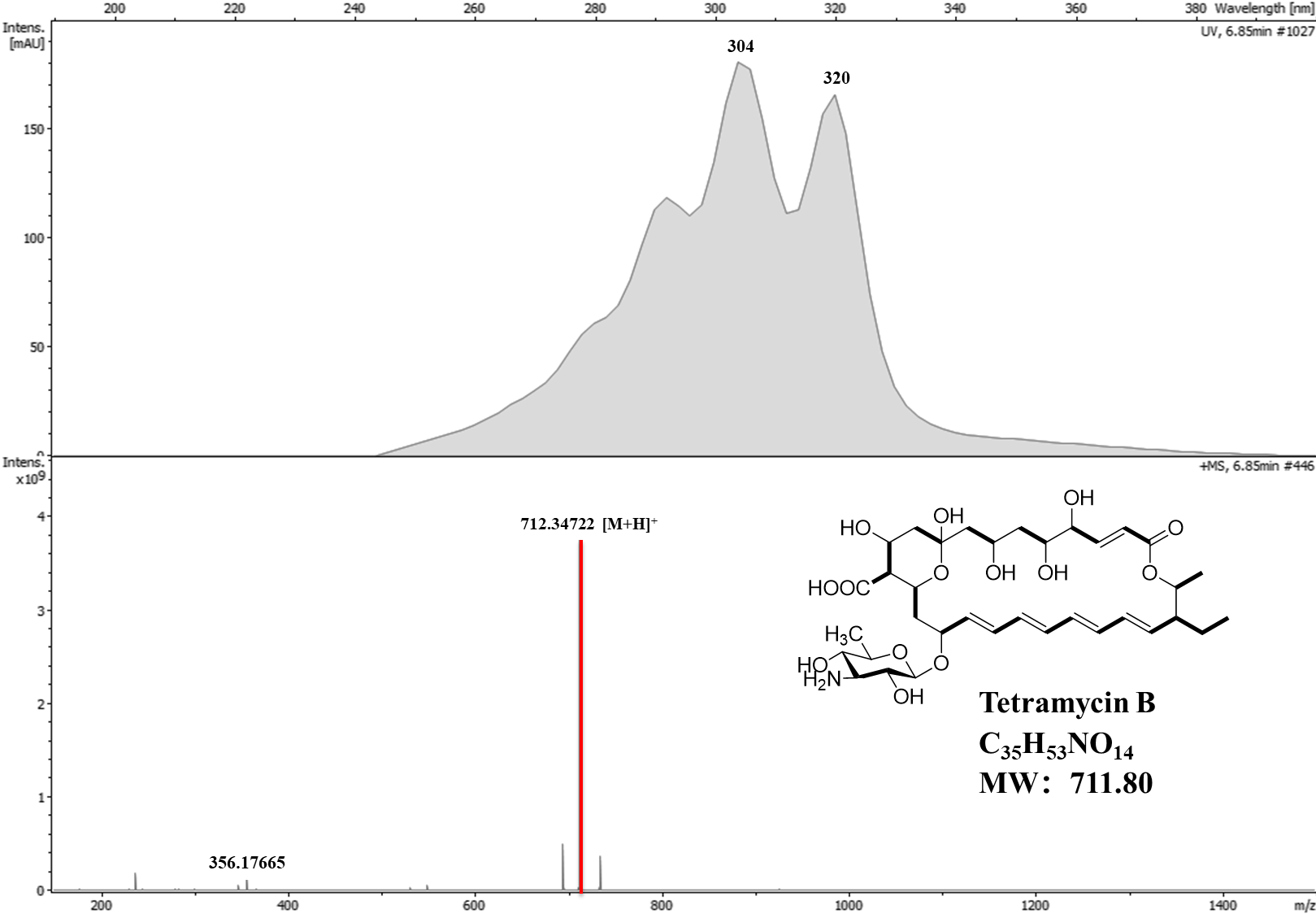
 Figure S2b**

**
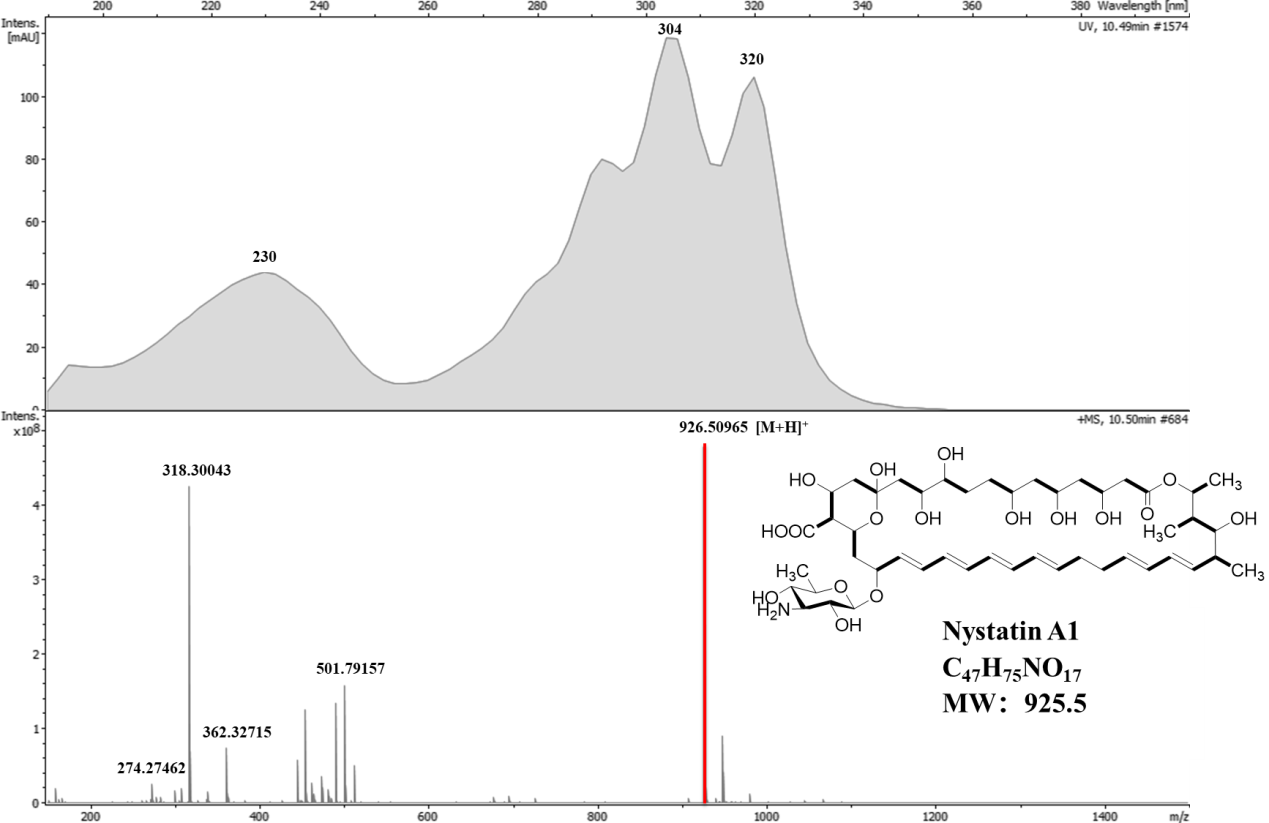
**

**Figure S2c**

**
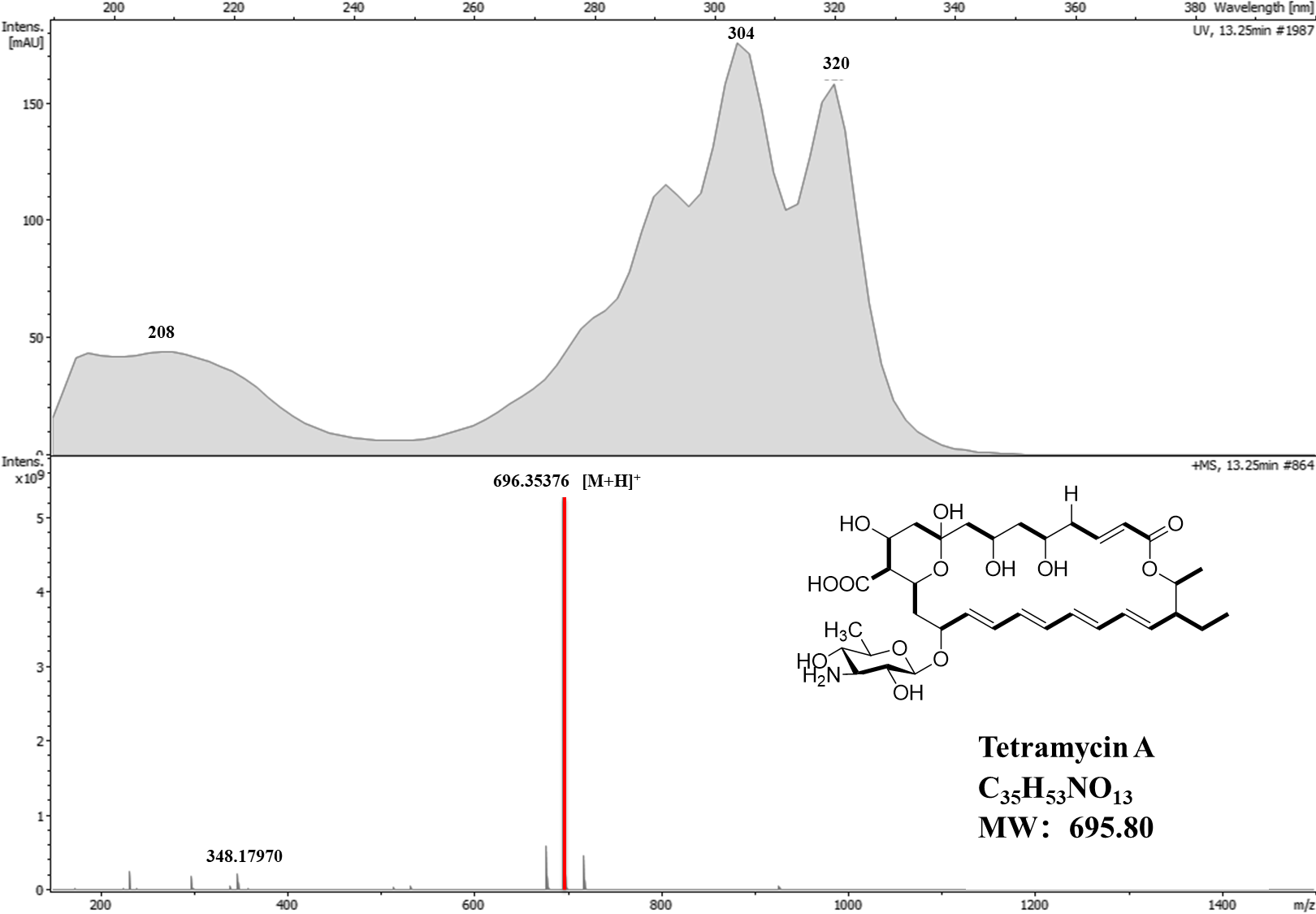
 Figure S2d**
