# Supplementary material for: Optimization of tetramycin production in Streptomyces ahygroscopicus S91
Source: J Biol Eng. 2021 May 22;15:16. doi: 10.1186/s13036-021-00267-4 (PMC8141235; doi:10.1186/s13036-021-00267-4)
Supplement: Supplementary file 3 — Additional file 3: Figure S3. Inactivation of nysB in S.ahygroscopicus S91. a. Construction of the recombinant plamid pDNB; b. Double crossover validation of the recombinant strain S91-ΔNB; c.Verification of sequencing in the recombinant strain S91-ΔNB. [file 13036_2021_267_MOESM3_ESM.docx]

**Figure S3 Descriptions**

**Fig. S3** Inactivation of *nys*B in *S.ahygroscopicus* S91

a. Construction of the recombinant plamid pDNB; b. Double crossover validation of the recombinant strain S91-ΔNB; c.Verification of sequencing in the recombinant strain S91-ΔNB.

**Figure S3a**

**
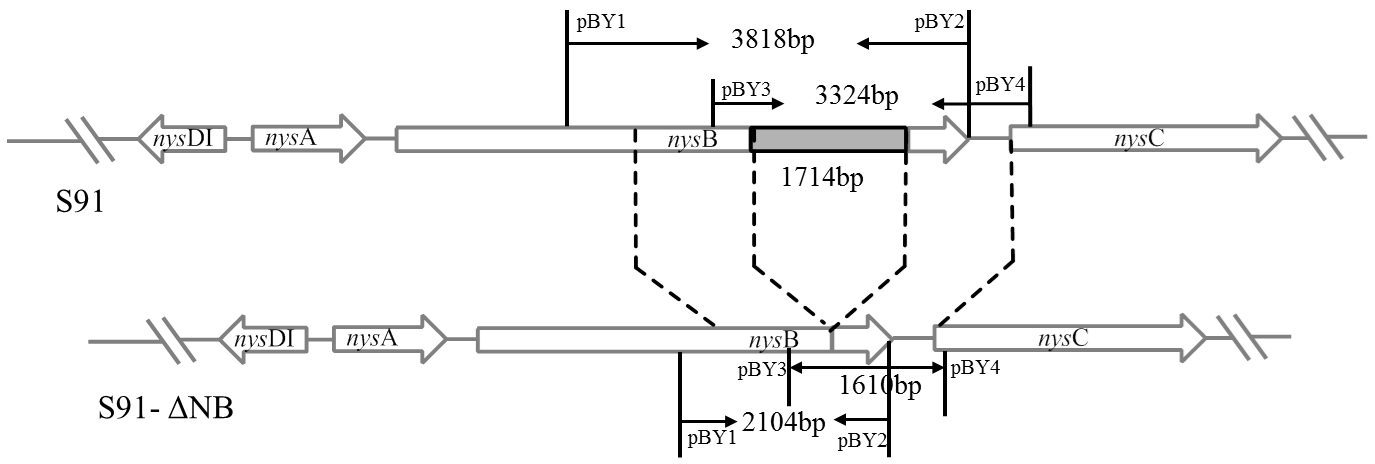
**

**Figure S3b**

**Figure S3c**
