# Supplementary material for: Optimization of tetramycin production in Streptomyces ahygroscopicus S91
Source: J Biol Eng. 2021 May 22;15:16. doi: 10.1186/s13036-021-00267-4 (PMC8141235; doi:10.1186/s13036-021-00267-4)
Supplement: Supplementary file 4 — Additional file 4: Figure S4. Inactivation of ttmD in S.ahygroscopicus S91-ΔNB. a. Construction of the recombinant plasmid pDTD; b. Double crossover validation of the recombinant strain S91-ΔNBΔTD; c. Verification of sequencing in the recombinant strain S91-ΔNBΔTD. [file 13036_2021_267_MOESM4_ESM.docx]

**Figure S4 Descriptions**

**Fig. S4** Inactivation of *ttm*D in *S.ahygroscopicus* S91-ΔNB

a. Construction of the recombinant plasmid pDTD; b. Double crossover validation of the recombinant strain S91-ΔNBΔTD; c. Verification of sequencing in the recombinant strain S91-ΔNBΔTD.

**Figure S4a**

**
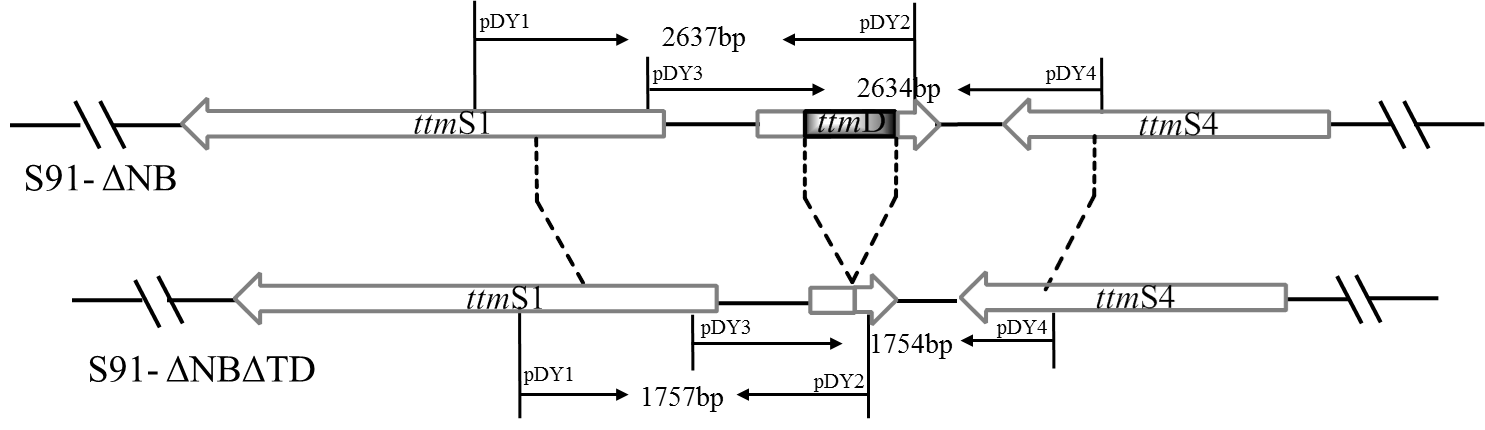
**

**Figure S4b**

**Figure S4c**
