# Supplementary material for: Optimization of tetramycin production in Streptomyces ahygroscopicus S91
Source: J Biol Eng. 2021 May 22;15:16. doi: 10.1186/s13036-021-00267-4 (PMC8141235; doi:10.1186/s13036-021-00267-4)
Supplement: Supplementary file 5 — Additional file 5: Figure S5. Cloning and overexpression of ttmRIV. a. Construction of the recombinant plamid pETRIV; b. PCR analysis of the recombinant strain S91-ΔNBΔTD::TRIV, M. DL2000 (2.0k, 1.0k, 0.75k, 0.5k, 0.25k, 0.1k), 1. S91-ΔNBΔTD::pETRIV/PB-1& TRIV-R (1.1k), 2. S91-ΔNBΔTD/PB-1&TRIV-R, 3. S91-ΔNBΔTD::pSET152/PB-1&TRIV-R. [file 13036_2021_267_MOESM5_ESM.docx]

**Figure S5 Descriptions**

**Fig. S5** Cloning and overexpression of *ttm*RIV

a. Construction of the recombinant plamid pETRIV; b. PCR analysis of the recombinant strain S91-ΔNBΔTD::TRIV, M. DL2000 (2.0k, 1.0k, 0.75k, 0.5k, 0.25k, 0.1k), 1. S91-ΔNBΔTD::pETRIV/PB-1& TRIV-R (1.1k), 2. S91-ΔNBΔTD/PB-1&TRIV-R, 3. S91-ΔNBΔTD::pSET152/PB-1&TRIV-R.

**
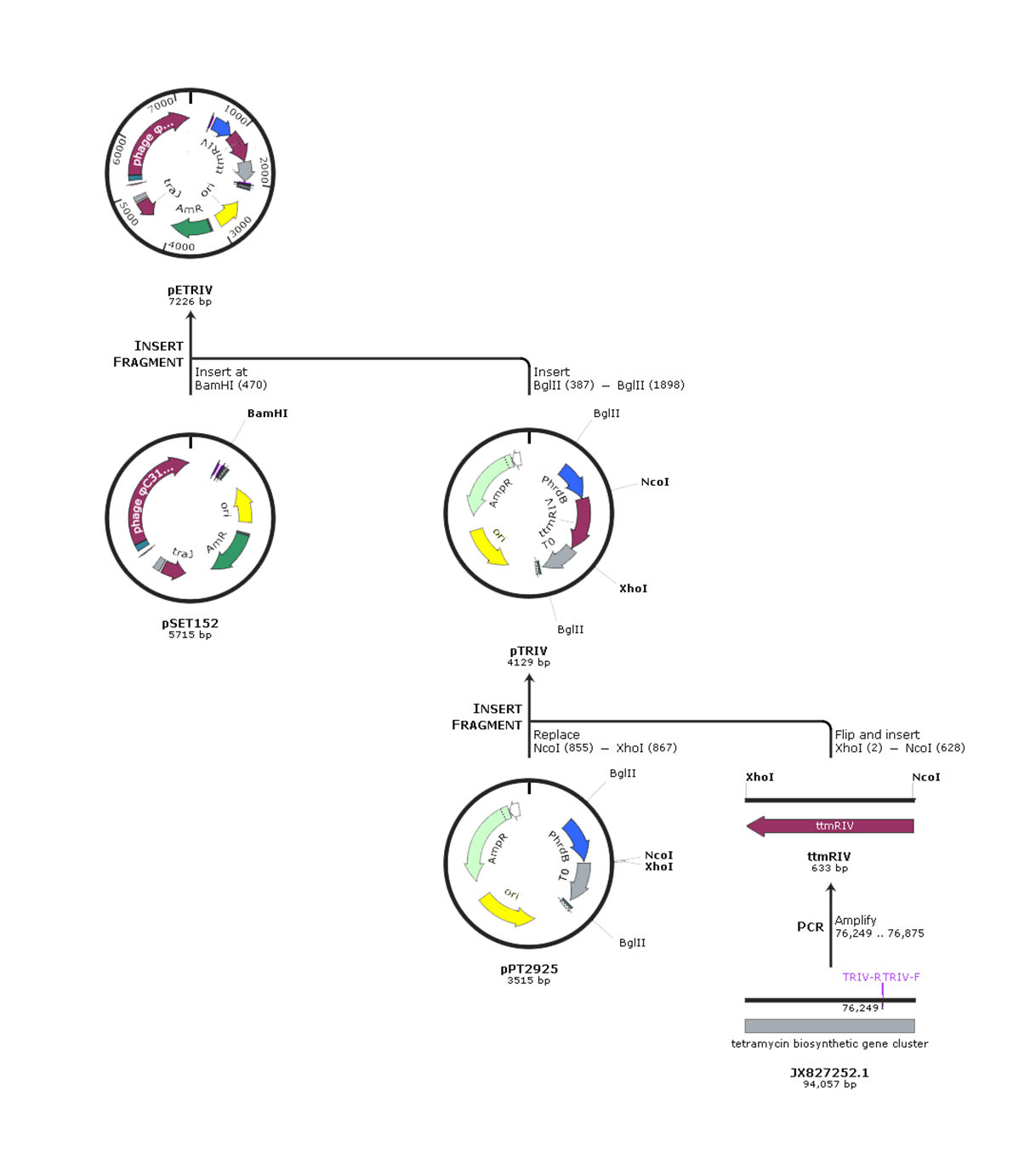
**

**Figure S5a**

**
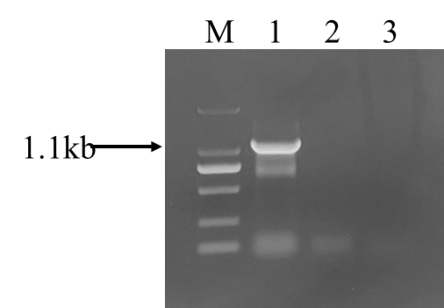
**

**Figure S5b**
