# Supplementary material for: Optimization of tetramycin production in Streptomyces ahygroscopicus S91
Source: J Biol Eng. 2021 May 22;15:16. doi: 10.1186/s13036-021-00267-4 (PMC8141235; doi:10.1186/s13036-021-00267-4)
Supplement: Supplementary file 6 — Additional file 6: Figure S6. Cloning and overexpression of ttmD. a. Construction of the recombinant plamid p2ETD; b. PCR analysis of the multicope ttmD recombinant strains, M. DL2000, 1. S91-ΔNB::pETD/PB-1&TD-R (1.7k), 2. S91-ΔNB::p2ETD/PB-1&TD-R (1.7k), 3. S91-ΔNB::p3ETD/PB-1&TD-R (1.7k), 4. S91-ΔNB/PB-1&TD-R, 5. S91-ΔNB::pSET152/PB-1&TD-R. [file 13036_2021_267_MOESM6_ESM.docx]

**Figure S6 Descriptions**

**Fig. S6** Cloning and overexpression of *ttm*D

a. Construction of the recombinant plamid p2ETD; b. PCR analysis of the multicope *ttm*D recombinant strains, M. DL2000, 1. S91-ΔNB::pETD/PB-1&TD-R (1.7k),

2. S91-ΔNB::p2ETD/PB-1&TD-R (1.7k), 3. S91-ΔNB::p3ETD/PB-1&TD-R (1.7k),

4. S91-ΔNB/PB-1&TD-R, 5. S91-ΔNB::pSET152/PB-1&TD-R.


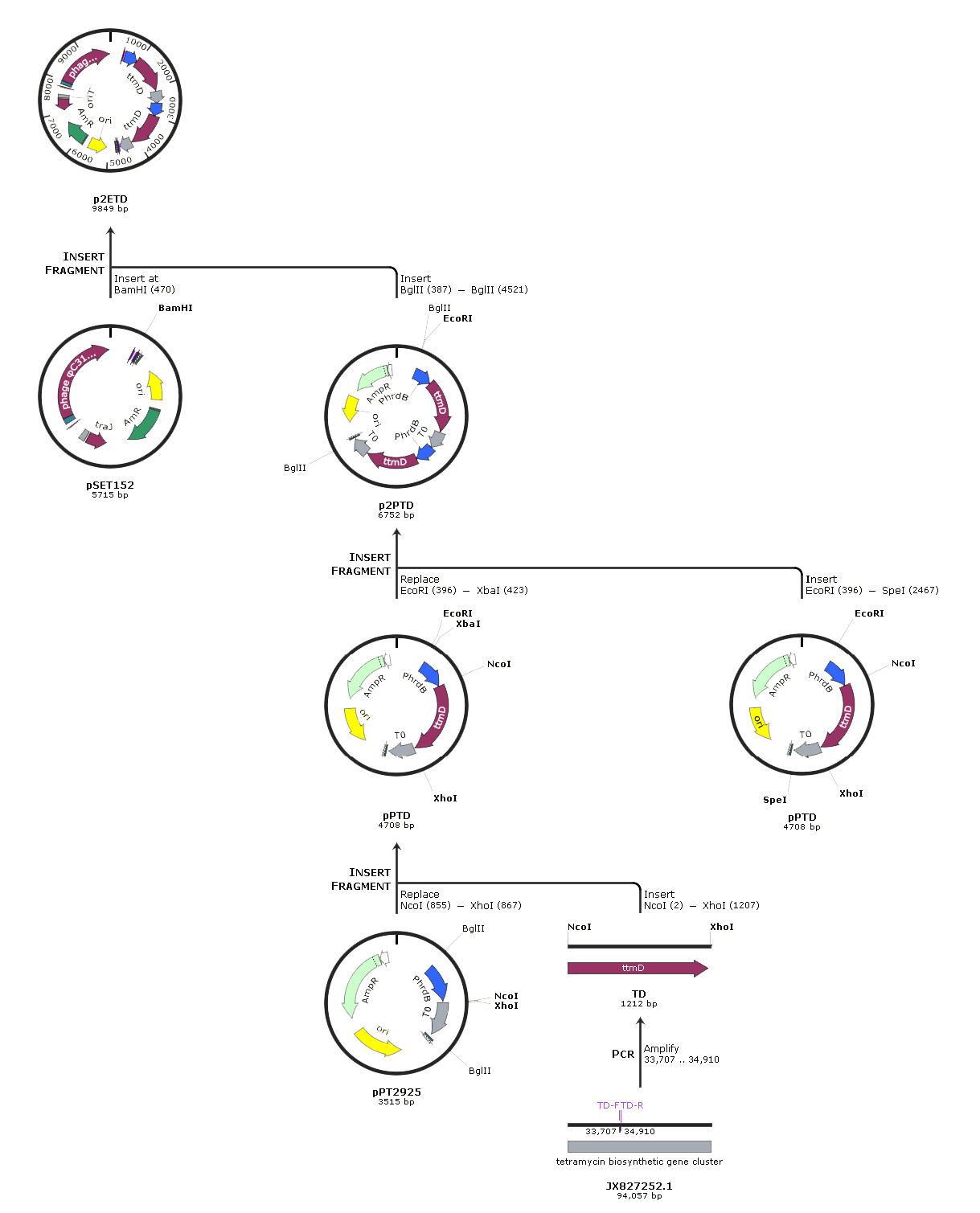


**Figure S6a**

**
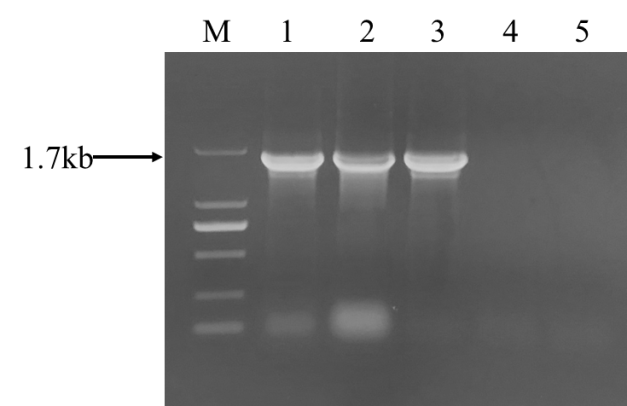
**

**Figure S6b**
