# Supplementary material for: Optimization of tetramycin production in Streptomyces ahygroscopicus S91
Source: J Biol Eng. 2021 May 22;15:16. doi: 10.1186/s13036-021-00267-4 (PMC8141235; doi:10.1186/s13036-021-00267-4)
Supplement: Supplementary file 7 — Additional file 7: Table S1. Production analysis in S.ahygroscopicus S91 and its mutants. [file 13036_2021_267_MOESM7_ESM.docx]

**Table S1** Production analysis in *S.ahygroscopicus* S91 and its mutants

| Strain | TA content (%) | TA yield  (mg·L^-1^) | TB content  (%) | TB yield (mg·L^-1^) |
| --- | --- | --- | --- | --- |
| S91 | 35.61 ± 1.80 | 323.75 ± 22.98 | 21.31 ± 1.18 | 193.74 ± 13.75 |
| S91-ΔNB | 51.59 ± 3.90 | 443.22 ± 29.19 | 26.64 ± 1.97 | 224.04 ± 16.57 |
| S91-ΔNBΔTD | 79.01 ± 1.83 | 888.62 ± 111.98 | - | - |
| S91-ΔNBΔTD  ::pSET152 | 72.63 ± 6.63 | 733.65 ± 79.19 | - | - |
| S91-ΔNBΔTD::RIV | 79.86 ± 1.94 | 1090.49 ± 136.65 | - | - |
| S91-ΔNB::pSET152 | 48.86 ± 0.72 | 325.22 ± 25.96 | 25.27 ± 0.83 | 163.39 ± 5.37 |
| S91-ΔNB::TD | 36.32 ± 1.40 | 289.41 ± 31.07 | 36.92 ± 1.94 | 294.33 ± 15.47 |
| S91-ΔNB::2TD | 20.01 ± 2.00 | 137.62 ± 26.82 | 51.63 ± 2.06 | 370.79 ± 14.79 |
| S91-ΔNB::3TD | 33.52 ± 1.38 | 397.02 ± 46.19 | 44.76 ± 1.90 | 533.59 ± 22.65 |

Values are means ± standard deviations.
