# Supplementary material for: Optimization of tetramycin production in Streptomyces ahygroscopicus S91
Source: J Biol Eng. 2021 May 22;15:16. doi: 10.1186/s13036-021-00267-4 (PMC8141235; doi:10.1186/s13036-021-00267-4)
Supplement: Supplementary file 8 — Additional file 8: Table S2. Strains, plasmids and primers used in this study. [file 13036_2021_267_MOESM8_ESM.docx]

**Table S2** Strains, plasmids and primers used in this study

| Strains, plasmids, primers | Relevant properties | Source or reference |
| --- | --- | --- |
| *E.coli* Top10 | Cloning host | Invitrogen |
| *E.coli* ET12567  （pUZ8002） | For intergenic conjugation, Kan^R^, Cl^R^ | **[1]** |
| *S.ahygroscopicus* S91 | Initial strain for tetramycin A, tetramycin B, nystain | CGMCC 4.7082 |
| S91-ΔNB | Nystatin disruption mutant | This study |
| S91-ΔNBΔTD | Nystatin and tetramycin B disruption mutant | This study |
| S91-ΔNBΔTD::TRIV | Nystatin and tetramycin B disruption mutant with  two copies of *ttm*RIV | This study |
| S91-△NB::TD | Nystatin disruption mutant with two copies of *ttm*D | This study |
| S91-△NB::2TD | Nystatin disruption mutant with three copies of *ttm*D | This study |
| S91-△NB::3TD | Nystatin disruption mutant with four copies of *ttm*D | This study |
| pKC1139 | Thermo-sensitive, Am^R^, *oriT* RK2, *ori* psG5 | **[2]** |
| pSET152 | Am^R^, *oriT* RK2, *int* φC31, *attP* φC31, *ori* pUC18 | **[3]** |
| pPT2925 | Clone plasmid, Amp^R^, *P_hrdB_*, *T_0_* | Lab construction |
| pDNB | Construct for nystatin disruption | This study |
| pDTD | Construct for *ttm*D disruption | This study |
| pETRIV | pSET152 with *ttm*RIV | This study |
| pETD | pSET152 with *ttm*D | This study |
| p2ETD | pSET152 with two copies of *ttm*D | This study |
| p3ETD | pSET152 with three copies of *ttm*D | This study |
| NB-UF | AAGCTTCGGTGAAGTCCAACATCGG |  |
| NB-UR | TCTAGACGGCCTCTTGGGTCTCCTG |  |
| NB-DF | TCTAGACGTGACCCTCATGGACTGG |  |
| NB-DR | GGATCCCGGTGAAGGAGACGAACGGG |  |
| pBY1 | ACCGGCACGAAGCTGGG |  |
| pBY2 | TCGGAGTCGGCGAGTTTCA |  |
| pBY3 | CGATCCAGGAGACCCAAGAG |  |
| pBY4 | GTCCTGGTTGATGGCGGAAC |  |
| TD-UF | GAATTCCGGGCGTTGCTGTCCGTAC |  |
| TD-UR | TCTAGAGATGCGTTCGTCGTTCAGCAGCT |  |
| TD-DF | TCTAGACGGTCTTCACCCAGTTGTTCACG |  |
| TD-DR | AAGCTTCGGCACTTCCTCACCTCCATCA |  |
| pDY1 | CGCGTCCATGTGCAGGGTCT |  |
| pDY2 | GCCTTCGTTCTCCGCCAGTT |  |
| pDY3 | TGCTTCTGCTCCGTCTCGTCCAATT |  |
| pDY4 | ACTCGCTGACCTCCGTCGAACTGC |  |
| TRIV-F | CCATGGTGGATCCCGCTCTGAC |  |
| TRIV-R | CTCGAGTTACTTGATGAAGTCGTCCA |  |
| PB-1 | GAATTCCGCCTTCCGCCGGAACG |  |
| PB-RT-1 | GTGTCGGCCAGCACATC |  |
| PB-RT-2 | CACTGGGTTGGCGGAATC |  |
| RIV-RT-1 | CTCCAGAATTCGCGCGTCGATC |  |
| RIV-RT-2 | CGGCCGACCGGTGACGTGTTC |  |
| TD-F | CCATGGCCTCCCCCCACCGTGATCT |  |
| TD-R | CTCGAGGCGTCGCGCCGACTACC |  |
| TD-RT-1 | CTCCTGCGGGACCTCCAG |  |
| TD-RT-2 | CGGGTCCGGATGCGAATG |  |
